# Alternative RNA Splicing Expands the Developmental Plasticity of Flowering Transition

**DOI:** 10.3389/fpls.2019.00606

**Published:** 2019-05-08

**Authors:** Young-Joon Park, June-Hee Lee, Jae Young Kim, Chung-Mo Park

**Affiliations:** ^1^Department of Chemistry, Seoul National University, Seoul, South Korea; ^2^Plant Genomics and Breeding Institute, Seoul National University, Seoul, South Korea

**Keywords:** alternative splicing, flowering, photoperiod, temperature, developmental aging

## Abstract

Precise control of the developmental phase transitions, which ranges from seed germination to flowering induction and senescence, is essential for propagation and reproductive success in plants. Flowering induction represents the vegetative-to-reproductive phase transition. An extensive array of genes controlling the flowering transition has been identified, and signaling pathways that incorporate endogenous and environmental cues into the developmental phase transition have been explored in various plant species. Notably, recent accumulating evidence indicate that multiple transcripts are often produced from many of the flowering time genes via alternative RNA splicing, which is known to diversify the transcriptomes and proteasomes in eukaryotes. It is particularly interesting that some alternatively spliced protein isoforms, including COβ and FT2β, function differentially from or even act as competitive inhibitors of the corresponding functional proteins by forming non-functional heterodimers. The alternative splicing events of the flowering time genes are modulated by developmental and environmental signals. It is thus necessary to elucidate molecular schemes controlling alternative splicing and functional characterization of splice protein variants for understanding how genetic diversity and developmental plasticity of the flowering transition are achieved in optimizing the time of flowering under changing climates. In this review, we present current knowledge on the alternative splicing-driven control of flowering time. In addition, we discuss physiological and biochemical importance of the alternative splicing events that occur during the flowering transition as a molecular means of enhancing plant adaptation capabilities.

## Introduction

Plants coordinately incorporate both exogenous and endogenous signals to fine-tune the timing of flowering transition under changing environments, among which the effects of light and temperature have been most extensively studied. Therefore, plants have evolved versatile mechanisms to accurately monitor seasonal changes in photoperiod and temperatures (Duncan et al., 2015; Blackman, 2017). Multiple flowering time genes are differentially affected by various environmental conditions. Endogenous cues, including plant aging signals and circadian rhythms, also affect the timing of flowering transition (Wang, 2014; Shim et al., 2017). It is now evident that the flowering transition is tightly regulated through a complex network of flowering genetic pathways, each monitoring distinct internal and external changes.

The flowering time genes are regulated through diverse molecular and biochemical mechanisms, such as transcriptional, post-transcriptional, and protein-level controls (Liu et al., 2008; Wang et al., 2016). They are also modulated by epigenetic mechanisms (Kim et al., 2004). Accumulating evidence in recent years indicate that alternative splicing, a versatile molecular process that produces multiple transcripts from a single gene and thus is capable of expanding the transcriptomes and proteomes, plays a critical role in flowering time control (Eckardt, 2002). Notably, the alternatively spliced protein isoforms either promote or suppress the corresponding functional proteins, depending on developmental and environmental conditions (Seo et al., 2012). Therefore, we believe that unraveling the functional roles of alternative splicing events would further expand the functional repertoire of the previously identified flowering time genes, especially in response to fluctuating external conditions.

In this review, we summarize recent advances in understanding the functional roles of alternative splicing events during flowering transition. Physiological and mechanistic relevance of the alternative splicing events are also discussed in terms of the developmental plasticity of flowering time control.

## Alternative Splicing of Photoperiodic Flowering Genes

Daylength information is a central determinant of photoperiodic flowering, and genes and molecular mechanisms underlying the photoperiod-dependent flowering induction have been characterized in many plant species (Sawa et al., 2007; Song et al., 2014; Lee et al., 2017). The floral activator CONSTANS (CO) plays a crucial role in photoperiodic flowering (Shim et al., 2017). It has been reported that FLAVIN-BINDING, KELCH REPEAT, F BOX 1 (FKF1) and GIGANTEA (GI) proteins interact with each other under long days (LDs), while this interaction does not occur under short days (SDs) (Sawa et al., 2007). The FKF1-GI complex suppresses the function of CYCLING DOF FACTOR 1 (CDF1), which acts as a CO repressor, thereby inducing the transcription of *CO* mainly under LDs.

In addition to the transcriptional control of *CO*, the CO function is also regulated at the protein level. It is known that CO proteins undergo post-translational modifications (Liu et al., 2008; Jung et al., 2012; Lazaro et al., 2012, 2015). On the other hand, the E3 ubiquitin ligase HIGH EXPRESSION OF OSMOTICALLY RESPONSIVE GENES 1 (HOS1) polyubiquitinates CO, leading to a controlled degradation of CO proteins in a red light-dependent manner (Lazaro et al., 2012, 2015). Meanwhile, in the dark, the E3 ubiquitin ligase CONSTITUTIVE PHOTOMORPHOGENIC 1 (COP1) targets the CO proteins (Liu et al., 2008). Notably, FKF1 conveys blue light information into the ubiquitin-proteasome system to enhance the CO protein stability, thus triggering the onset of photoperiodic flowering (Song et al., 2012; Lee et al., 2017).

A previous study has shown that CO undergoes alternative splicing, producing two protein isoforms: the full-size, physiologically functional COα, which is equivalent to the well-characterized CO flowering promoter, and the C-terminally truncated COβ (Gil et al., 2017). All the previous functional studies on CO have been performed with the COα protein isoform (Liu et al., 2008; Jung et al., 2012; Lazaro et al., 2012, 2015; Song et al., 2012, 2014; Wang et al., 2014, 2016), and, as a result, the potential importance of its alternative splicing in photoperiodic flowering has been elusive until recently. The COα form contains both the B-box (BBX) and CCT (for CONSTANS, CONSTANS-LIKE, TOC1) domains, whereas the C-terminally truncated COβ form lacks the CCT domain.

**FIGURE 1:**
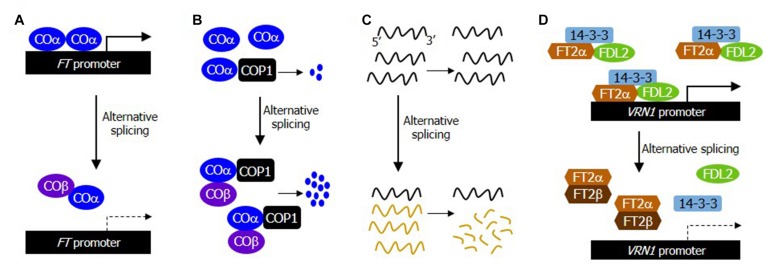
Alternative RNA splicing regulates the timing of flowering transition through diverse regulatory mechanisms. In response to developmental and environmental cues, numerous flowering time genes undergo alternative splicing. The alternatively spliced RNA isoforms are regulated either at the RNA level through the NMD pathway or translated to multiple protein products. The alternatively spliced protein variants modulate the stability or function of different variants by forming heterodimers. They also interact differentially with their interacting partners in a competitive manner. **(A)** Attenuation of the DNA binding affinity of COα by forming non-DNA-binding, COα-COβ heterodimers in photoperiodic flowering. While the COα-COα homodimers are able to efficiently bind to DNA, the COα-COβ heterodimers are excluded from DNA binding. **(B)** Facilitation of the interaction of COα with the COP1 E3 ubiquitin ligase by COβ in photoperiodic flowering. While COα monomers are poorly targeted by COP1, COβ facilitates the interaction of COα with COP1, resulting in an elevated ubiquitin-mediated degradation of the COα proteins. **(C)** Differential stabilization of alternatively spliced transcripts against NMD pathway in thermosensory flowering. The alternatively spliced transcripts are degraded through the NMD pathway. **(D)** Attenuation of the FT2α/FDL2/14-3-3 florigenic complex formation by FT2β in aging-induced flowering time control. The alternatively spliced protein isoform FT2β inhibits the formation of the FT2α-containing florigenic complexes by forming FT2α-FT2β heterodimers in Brachypodium.

In accordance with the notion that CO is a flowering activator, overexpression of COα accelerates flowering induction (Gil et al., 2017). Notably, transgenic plants overexpressing COβ exhibited late flowering, which is phenotypically similar to what is observed in CO-deficient mutants. In addition, the promotive effects of COα on flowering induction were compromised when COβ was co-expressed with COα, suggesting that COβ acts as a competitive inhibitor of COα. Transcription factors act typically as dimers to enhance their DNA binding affinity and specificity (Seo et al., 2011). It has been found that COβ attenuates the COα function by forming non-functional heterodimers, which have a significantly reduced DNA binding capability compared to that of the COα-COα homodimer (Figure 1A; Gil et al., 2017).

A critical question is how photoperiodic information is functionally linked with the alternative splicing event of CO. It has been observed that while the absolute level of *COα* transcripts is much higher than that of *COβ* transcripts, the relative ratio between the two RNA isoforms is unchanged during photoperiodic flowering (Gil et al., 2017). Interestingly, COβ protein is resistant to the ubiquitin-proteasome degradation. Meanwhile, the protein stability of COα is modulated in a complicated manner by a group of E3 ubiquitin ligases. COβ enhances the interaction of COα with HOS1 and COP1, while COβ suppressed the interaction of COα with FKF1, leading to a further destabilization of COα. Together, these observations indicate that CO is not a passive substrate of the E3 ubiquitin ligases. Instead, CO acts as a proactive regulator of its own protein accumulation by modulating its interactions with multiple E3 ubiquitin ligases in a coordinated manner during the induction of photoperiodic flowering (Figure 1B).

CO belongs to the BBX transcription factor family, which consists of 32 members in *Arabidopsis*. It has been reported that other BBX transcription factors, which are structurally similar to either COα or COβ, are also functionally linked with flowering time control (Wang, 2014), further supporting the functional relevance of alternative splicing for photoperiodic flowering. It is also interesting that the alternative splicing event of CO is not confined to *Arabidopsis*. A putative *Brachypodium* CO also undergoes alternative splicing, producing two protein isoforms: the full-size CO isoform and the C-terminally truncated CO isoform (Gil et al., 2017). Both *Arabidopsis* and *Brachypodium* are LD plants, flowering early during LDs. It will be interesting to examine whether the CO alternative splicing is a conserved molecular event in all LD plants.

## Alternative Splicing Events During Thermosensory Flowering

Global warming, a gradual increase of the average global temperature, is widely considered as a serious environmental concern in recent decades. It is well-known that even small changes in ambient temperatures profoundly affect the growth patterning and the timing of developmental transitions in plants, and thus studies on genes and associated molecular mechanisms underlying plant temperature adaptation attracts particular attention in recent years (Quint et al., 2016; Park et al., 2017).

It has been documented for a long time that plants are capable of coping with extreme temperature stress, such as heat and freezing (Ding et al., 2015; Han et al., 2019). Numerous genes and stress adaptation mechanisms have been functionally characterized (Chinnusamy et al., 2007; Ohama et al., 2017). On the other hand, plants often encounter mild temperature changes rather than temperature extremes in natural habitats. In response to changes in ambient temperatures, plants exhibit multiple distinct phenotypes, such as stem elongation, elevation of leaf hyponasty, and acceleration of flowering initiation, which are collectively termed thermomorphogenesis (Koini et al., 2009; Quint et al., 2016; Park et al., 2019). It is known that the thermomorphogenic process is distinct from temperature stress responses and these two thermal responses are regulated by different sets of genes and regulatory mechanisms (Quint et al., 2016). Among the pleiotropic thermomorphogenic phenotypes, the thermal control of flowering initiation has been extensively studied because of its direct association with reproductive success and crop productivity in temperate areas (Lee et al., 2013).

FLOWERING LOCUS M (FLM) is a MADS box transcription factor functioning as a floral repressor (Sureshkumar et al., 2016). It has been observed that temperature-responsive flowering is nearly diminished in FLM-deficient mutants (Balasubramanian et al., 2006), showing that FLM is involved in thermosensory flowering. A critical question is how temperature signals modulate the FLM function in controlling thermosensory flowering. Interestingly, FLM undergoes alternative splicing, producing multiple *FLM* transcripts (Sureshkumar et al., 2016). In addition, its alternative splicing pattern is altered in response to temperature changes, supporting that the alternative splicing process of *FLM* is a critical constituent of temperature-sensitive timing of flowering. A question is how the temperature-mediated production of multiple transcripts is associated with the timing of thermosensory flowering.

Eukaryotes have evolved a molecular surveillance system to remove any potential defects in gene expression by eliminating non-functional or damaged mRNA transcripts, a molecular machinery often termed nonsense-mediated mRNA decay (NMD) (Karousis et al., 2016). In this sense, it is apparent that gene expression is regulated by the NMD-mediated mRNA degradation as well as mRNA transcription. Interestingly, alternatively spliced *FLM* transcripts are more rapidly degraded by the NMD pathway at warm temperatures, while they are relatively stable at low ambient temperatures (Figure 1C; Sureshkumar et al., 2016). Consistently, temperature-responsive flowering is compromised in *Arabidopsis* mutants that are defective in the NMD pathway. The differential sensitivity of the alternatively spliced *FLM* transcripts to the NMD pathway at different temperatures illustrates a pivotal role of the NMD-mediated surveillance system in thermosensory flowering.

In addition to the NMD-mediated degradation of the alternatively spliced transcripts, alternative splicing might provide an additional molecular mechanism that regulates FLM function. FLM physically interacts with another MADS box transcription factor, SHORT VEGETATIVE PHASE (SVP), which also functions as a floral repressor (Lee et al., 2013). Both *flm* and *svp* mutants are insensitive to changes in ambient temperatures, showing that SVP and FLM are tightly linked with thermosensory flowering.

It is known that warm temperatures reduce the binding of the SVP transcription factor to the promoter of *FLOWERING LOCUS T* (*FT*) gene (Lee et al., 2007). Interestingly, the temperature-sensitive binding of SVP to DNA is abolished in *flm* mutant backgrounds (Lee et al., 2013), indicating that FLM facilitates the DNA binding ability of SVP. FLM undergoes alternative splicing, producing multiple protein isoforms, such as FLMβ and FLMδ. FLMβ is the functional floral repressor, whereas FLMδ is one of the alternatively spliced protein variants and has a reduced DNA-binding capability (Lee et al., 2013; Posé et al., 2013). SVP interact efficiently with both FLMβ and FLMδ. However, the SVP- FLMδ complex does not efficiently bind to the target promoter sequence, suggesting a mutually competitive inhibition between the FLM protein isoforms. While overexpression of *FLMδ* promotes flowering, this model has been proven to be inappropriate for explaining temperature-responsive flowering in *Arabidopsis* (Sureshkumar et al., 2016; Capovilla et al., 2017; Lutz et al., 2017). It still remains to be elucidated whether and how FLMδ contributes to flowering transition.

Most studies on alternative splicing utilize gene overexpressing systems to maximize the effects of individual alternatively spliced RNA or protein variants. However, the phenotypes of the resultant transgenic plants do not necessarily provide any information as to the endogenous effects of alternative splicing. Care should also be taken when interpreting the phenotypes of transgenic plants using endogenous promoters because of the positional effects of the gene insertion into plant genomes. An alternative approach is the CRISPR/Cas9-mediated genome editing system, a powerful technology for studying the effects of alternative splicing in that it is readily applicable to introducing a mutation into a specific splice site so that the patterns of an alternative splicing event are precisely engineered. Indeed, the genome editing system has been applied successfully to generate plants that produce only FLMβ but not FLMδ and *vice versa* (Capovilla et al., 2017).

Genomic *FLM* gene-engineered plants, which lack FLMβ production, flower earlier than wild-type plants, but not earlier than FLM-defective mutants. In addition, plants lacking FLMδ production flower later than wild-type plants, but not later than FLM-overexpressing plants (Capovilla et al., 2017). Therefore, it is likely that the negative regulatory effect of FLMδ is not dominant in wild-type plants. Collectively, these observations indicate that alternative splicing of FLM is a critical molecular device for the FLM-mediated control of thermoresponsive flowering.

In addition to FLM, multiple regulators of flowering timing undergo alternative splicing. It has been reported that trimethylated histone H3 at lysine 36 (*H3K36me3*), a marker of active gene transcription, is enriched in genes undergoing alternative splicing in mammals (Zhou et al., 2014). Chromatin immunoprecipitation assays using *Arabidopsis* plants exposed to different ambient temperatures have shown that H3K36me3 is enriched in the genomic sequence regions harboring flowering time genes and the H3K36me3-enriched regions are broader at warm temperatures (Pajoro et al., 2017). These observations support that temperature-responsive epigenetic control is intimately linked with the effects of ambient temperatures on alternative splicing.

H3K36me3 is directed by histone methyltransferases, SET DOMAIN GROUP 8 (SDG8) and SDG26, in *Arabidopsis* (Xu et al., 2008). Notably, the thermo-responsive alternative splicing of flowering time genes is disturbed in SDG-deficient mutants (Pajoro et al., 2017), further supporting the notion that temperature-responsive epigenetic control by H3K36me3 is tightly associated with alternative splicing events. Consistent with this observation, the flowering of *sdg* mutants are less sensitive to ambient temperatures. Taken together, these observations indicate that temperature-induced epigenetic modifications, such as H3K36me3, mediate the thermo-responsive alternative splicing of flowering time genes.

An ultimate question in the field is how plant temperature-sensing mechanisms affect the alternative splicing events of flowering time regulators. There have been studies aimed to identify such thermosensors in plants (Jung et al., 2016; Legris et al., 2016). The best characterized is the red/far-red light-sensing phytochrome photoreceptors, which also function as thermosensors in *Arabidopsis* (Jung et al., 2016; Legris et al., 2016). It is known that photoconversion of the physiologically activated Pfr form to the inactive Pr form is accelerated at warm temperature (Jung et al., 2016; Legris et al., 2016). Notably, the phytochromes have been implicated in the red light-dependent alternative splicing process (Shikata et al., 2014). It will be interesting to examine whether the phytochromes or any putative thermosensors are responsible for the alternative splicing of genes involved in flowering time during thermosensory flowering.

## Alternative Splicing During Developmental Control of Flowering

In the juvenile vegetative phase, plants are recalcitrant to floral activating signals, necessitating that plants must spend sufficient time in the vegetative phase to acquire reproductive competence. It is well-known that the mutually interacting microRNA156 (miR156)-miR172 pathway acts to provide developmental aging signals during flowering transition (Wang, 2014). Thus, miRNA-mediated degradation of target transcripts and their translational inhibition are regarded as a major molecular device for transmitting developmental aging signals.

It has been recently reported that alternative splicing plays an important role in the developmental control of flowering initiation in *Brachypodium distachyon*, a representative monocot model for studies on bioenergy grasses and cereal crops in the field (Brkljacic et al., 2011). In response to inductive photoperiodic signals, the FT florigen is produced in the leaves and transported to the shoot apical meristems (SAM) to induce flowering in *Arabidopsis* and other plant species (Jaeger and Wigge, 2007). FT interacts with 14-3-3 and FD proteins in SAM to promote flowering transition (Taoka et al., 2011). FT2 is a potential homolog of the *Arabidopsis* FT protein in *Brachypodium* (Qin et al., 2017). Interestingly, FT2 undergoes alternative splicing, producing the functional FT2α protein and the alternatively spliced FT2β protein (Qin et al., 2017).

Protein domain analysis revealed that the N-terminal region of FT2 harboring the phosphatidylethanolamine-binding protein (PEBP) domain is eliminated in the FT2β protein isoform, suggesting that FT2β lacks any mechanistic functions conferred by the PEBP domain. Notably, the alternatively spliced FT2β isoform is unable to interact with 14-3-3 and FD-LIKE 2 (FDL2) proteins, while FT2β is still able to interact with FT2α in *Brachypodium* (Qin et al., 2017). Extensive biochemical studies have shown that FT2β acts as a competitive inhibitor by attenuating the binding capability of FT2α with 14-3-3 and FDL2 proteins (Figure 1D). Consistent with this biochemical observations, the expression of *VERNALIZATION1* (*VRN1*) gene is significantly elevated in FT2β-specific knock-down plants (Qin et al., 2017).

What regulates the alternative splicing of FT2? It has been previously reported that the expression of *FT2* gene gradually increases as plant ages (Qin et al., 2017). The levels of both the *FT2*α and *FT2β* transcripts increase throughout developmental transitions. However, the *FT2β* transcripts are more abundant in young plants, while the *FT2α* transcripts are more abundant in old plants (Qin et al., 2017). These observations indicate that the alternative splicing patterns of FT2 is developmentally programmed to incorporate endogenous cues into flowering genetic pathways. The miR156-miR172 pathway is widely conserved in plants. It is worthy of examining whether and how miRNA-mediated developmental signals are linked with the alternative splicing event of FT2 in *Brachypodium*.

## Conclusion and Perspectives

Alternative splicing is wide spread in both plants and animals. In plants, it is involved in a variety of plant adaptation processes in response to aging and environmental stimuli. Many flowering time genes undergoes alternative splicing, and plants utilize this molecular devise to precisely control the onset of flowering under fluctuating environments.

It is notable that the proven and predicted mechanistic functions of alternatively spliced variants are quite diverse in flowering time control (Figure 1). For example, the alternatively spliced COβ variant interacts with the COα transcription factor to constitute non-DNA-binding heterodimers during photoperiodic flowering in *Arabidopsis*, while FT2β interferes with the protein-protein interactions between FT2α and FDL2 proteins during aging signal-induced flowering in *Brachypodium*. In addition, the COβ splice variant controls the accessibility of E3 ubiquitin ligases to COα. The stability of alternatively spliced transcripts are also targeted by the NMD pathway at the RNA level during thermosensory flowering. These observations indicate that alternative splicing provides a versatile regulatory system to incorporate multiple developmental and environmental signals into flowering genetic pathways to achieve fine-tuning of the time of flowering induction and maximal productivity.

**FIGURE 2:**
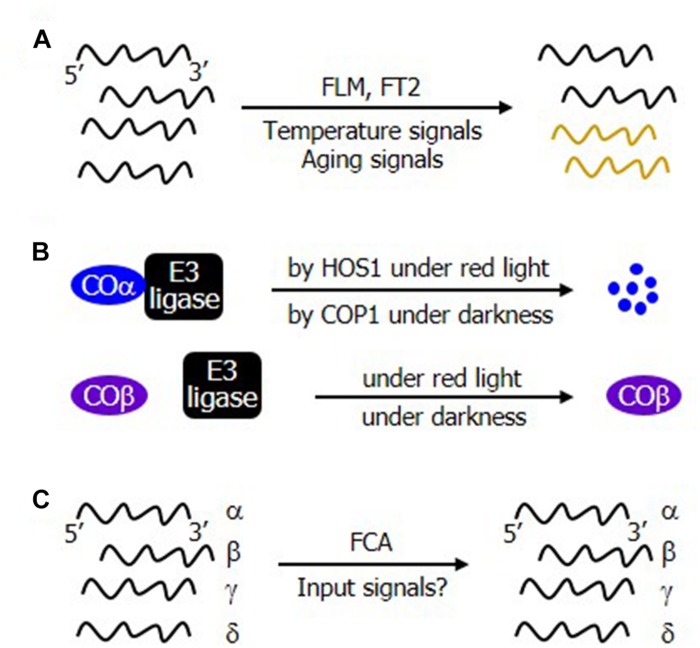
Regulatory modes of alternative splicing events during flowering transition. **(A)** Development and environmental regulation of alternative splicing. The alternative splicing patterns of the *Arabidopsis* FLM and the *Brachypodium* FT2 are modulated by temperature and plant aging signals, respectively, altering the molar ratios of alternatively spliced RNA isoforms. **(B)** Differential stability of the alternatively spliced protein isoforms of CO. While COα is targeted by the HOS1 and COP1 E3 ubiquitin ligases under red light and dark conditions, respectively, COβ is resistant to the ubiquitin-mediated degradation process. **(C)** Constitutive occurrence of alternative splicing in the autonomous flowering pathway. FCA is a central component of the autonomous flowering pathway. Its alternative splicing produces several transcript isoforms. It is currently unclear whether and how its alternative splicing is modulated by developmental cues or environmental stimuli.

It is also interesting that the patterns of alternative splicing are differentially regulated during flowering transition. For example, the relative ratio of the alternatively spliced transcripts of FLM in *Arabidopsis* and FT2 in *Brachypodium* is influenced by temperature and developmental aging signals, respectively (Figure 2A). Meanwhile, the ratio of the protein levels between COα and COβ changes during the day, while their transcript levels are unchanged (Figure 2B). Furthermore, the ratio of the alternatively spliced transcripts of a gene encoding the floral activator FLOWERING CONTROL LOCUS A (FCA), which functions via the autonomous flowering genetic pathway, is unchanged (Figure 2C; Macknight et al., 2002). It is evident that the regulatory modes of alternative splicing is quite diverse in plants.

Alternative splicing events are modulated by differential actions of splicing factors on primary transcripts. For example, SKI-INTERACTING PROTEIN (SKIP), which functions as a splicing factor, directly binds to the pre-mRNA of *SERRATED LEAVES AND EARLY FLOWERING* (*SEF*) to suppress its undesirable alternative splicing (Cui et al., 2017). Since SEF activates the transcription of *FLOWERING LOCUS C* (*FLC*), its transcript level is significantly low in SKIP-deficient mutants supporting that splicing factors play crucial roles during floral transition. In addition, it is possible that the activities of splicing factors are modulated by both external and internal cues through multiple molecular mechanisms, such as transcriptional and post-translational modifications and the formation of spliceosome complex (Xiao and Manley, 1998; Stankovic et al., 2016; Shang et al., 2017). It would be interesting to examine the functional relevance of the diversified patterns of alternative splicing in the developmental plasticity of flowering timing.

## Author Contributions

C-MP and Y-JP designed the concept and organization of the manuscript. C-MP and Y-JP wrote the manuscript with helps of J-HL and JK.

## Conflict of Interest Statement

The authors declare that the research was conducted in the absence of any commercial or financial relationships that could be construed as a potential conflict of interest.
